# The experience of patients and family caregivers during hospital-at-home in France

**DOI:** 10.1186/s12913-019-4295-7

**Published:** 2019-07-09

**Authors:** Hélène Rossinot, Odile Marquestaut, Matthieu de Stampa

**Affiliations:** 1 Publique Hôpitaux de Paris (AP-HP), Hospitalisation à Domicile (HAD), 14 rue Vésale, 75005 Paris, France; 2 Unité Mixte de Recherche (UMR) 1168 INSERM, UVSQ, Vieillissement et Maladies Chroniques (VIMA), Villejuif, France

**Keywords:** Hospital at home, Caregivers and patients at home, Quality of life, Healthcare organization

## Abstract

**Background:**

Public health policies tend to generalize the use of Hospital-At-Home (HAH) to answer the growing will of patients to be treated or to die at home. HAH is a model of care that provides acute-level services in the patient’s home with the interventions of variety of health care professionals. Relatives participate also in the interventions by helping for sick patients at home, but we lack data on the care of patients and caregivers in HAH. The aim of this study was to make an inventory of the experiences of patients and family caregivers in HAH.

**Methods:**

The research was qualitative using nineteen semi-directed interviews from nine patients and ten caregivers of one care unit of Greater Paris University Hospitals’ HAH, and the grounded theory was used to analyze the transcripts. Caregivers were also asked, after the interview, to fill in the Zarit Burden Inventory.

**Results:**

HAH remained mostly unknown for patients and caregivers before the admission proposition and the outlook of being admitted in HAH was perceived as positive, for both of them. Caregivers had a versatile role throughout HAH, leading to situations of suffering, but also had sources of support. The return home was considered satisfactory by both caregivers and patients, related to the quality of care and increased morale despite HAH’s organizational constraints. We noted an impact of HAH on the relationship between the patient and the caregiver(s), but caused by multiple factors: the fact that the care takes places at home, its consequences but also the disease itself.

**Conclusion:**

HAH strongly involved the patient’s caregiver(s) all along the process. HAH’s development necessitates to associate both patients and caregivers and to take into account their needs at every step. This study highlights the need to better assess the ability of the caregiver to cope with his or her relative in HAH with acute and subacute care at home.

**Electronic supplementary material:**

The online version of this article (10.1186/s12913-019-4295-7) contains supplementary material, which is available to authorized users.

## Background

Hospital at home (HAH) is expending all across the industrialized world. It answers the growing will of patients to be treated or to die at home [1], offers the possibility of a better satisfaction in their own environment [2, 3] while being economically advantageous for the health care system [1]. HAH provides acute or sub acute care at the patient home for a condition that would otherwise require a conventional hospitalization such as complex dressing, palliative care, intravenous treatment, chemotherapy or supportive care in cancer whatever the age of the patient [2]. HAH is the delivery of hospital ward level care in the patient’s home and replaces “real” hospital by ensuring the continuity of care for the patients it takes care of. HAH is also known as “hospital in the home”, “home hospitalization” and “early supported discharge” [3–5]. HAH substitutes itself for hospital care, may it be a full substitution (“admission avoidance HAH”) or a shortened hospitalization (early discharge from hospital) [6, 7]. HAH services are available 24 h a day and 7-days a week, and include care provided by an alliance of healthcare professionals such as nurses, assisting nurses, psychologists, physiotherapists but also general practitioners and in most cases the in-house medical coordinator [8, 9]. HAH services usually provide an holistic care approach but some of them focus on specialties such as surgery [10, 11], medical specialties [12, 13], rehabilitation medicine [14], geriatrics [15], respiratory diseases [16, 17] or infectious diseases [18]. They are different from community-nursing organizations, because of the active, advanced and intensive care provided and the variety of professionals required. HAH is a model of care that provides acute-level services in the patient home and can also in some cases be set up in a nursing home. HAH is a less expensive way than conventional hospitalization with an average cost of 198€/day in the French health system [19].

In most healthcare systems, when admitted to HAH, the patient is advised to have at least an informal caregiver. It can be a family member, a friend or a neighbor. The role of family caregivers in the care at home of their relatives increases more and more in occidental countries, and they provide the largest proportion of care [20, 21]. The burden for caregivers of informal care is the main factor of admission into nursing home of patients with dementia [22, 23]. Providing regular assistance to a disabled or an elderly person is considered a chronic stress for the caregiver [24, 25]. A growing body of research indicates that caregivers experience higher risk of health issues such as somatic illness, depression and cognitive decline [26–28]. Involving family caregivers both more and better in the patient’s care during HAH intervention is essential to increase the quality of care. But there is very few data on the experience of patients and their family caregiver during HAH and the existence or not of an impact of home **care** on their relationship, despite the fact that this mode of care is spreading. To our knowledge, no study has simultaneously involved the caregivers’ and patients’ points of views, although there is a need to develop a fuller understanding of the role they played.

This study’s objectives were to identify the point of view on care of family caregivers and patients’ relatives during the hospitalization at home, and to describe their relationship and its evolution during the time of the care. Our findings enabled us to understand more deeply the roles and interactions of the patients and the caregivers during at-home care and to better meet their needs in order to improve the quality of care and to reinforce the development of HAH all over the world.

## Methods

Given the lack of knowledge about the experience of patients and their family caregiver during HAH, a qualitative method was deemed appropriate using interviews from patients and caregivers of one care unit of Greater Paris University Hospitals’ HAH. We adopted an inductive strategy to investigate the roles and the relationships between patients and caregivers using a grounded theory approach in our analysis [29]. Caregivers were also asked, after the interview, to fill in the Zarit Burden Inventory. The aim was to compare the verbatim of the caregivers to their answers to the questionnaire and observe if the description of their situation obtained during the interviews matched the answers to enable us to reinforce our comprehension of the topic. Pseudonyms were used to maintain the anonymity of participants.

### Description of France’s HAH system and of the study location

HAH has been part of the French health system for several decades, but has risen to some prominence over the last 20 years. The legal recognition of HAH system goes back to the 1970s but HAH officially became an alternative to hospital inpatient care in 1991 [30]. Since 2009, HAH is a distinct choice of hospitalization in itself on the French territory [31]. Many HAH have a hospital status, others are considered as a department of a hospital. They are either public, private not-for-profit, or private for-profit and the first two categories are the most common (each of them represents 41% of all HAH care providers). In 2015, altogether French HAH delivered 4,629,254 bed days in 160,793 admissions to 105,008 patients with an average length of stay of 25 days. The activity of the ensemble of HAH reached 4.6% of the total of bed days in 2015 in France and reimbursements to all 308 french HAH institutions represented a total of 913 million Euros [32]. The State has set ambitious growth targets for HAH over the next few years. HAH’s mission is to “ensure, in the patient’s home, suffering from severe, complex and progressive disease(s), for a limited period of time, but revisable depending on the evolution of his health condition, continuous and coordinated medical and paramedical care that only a hospital facility can provide” [33]. Most of the HAH institutions on the French territory provide polyvalent care. Some specific interventions are allowed during HAH, such as: complex dressings, palliative care, complex nursing care and geriatric, enteral nutrition, intravenous treatment, post chemotherapy surveillance, respiratory care, parenteral nutrition, at-risk antenatal pregnancy and post-partum pathology management, chemotherapy, pain management and post-surgery management. Patients may have one or more conditions, accompanied or not by poor social and financial backgrounds. HAH also attempts to introduce initiatives in physical support and in patient and caregiver therapeutic education that will persist beyond discharge.

Arrangements with hospitals and community-based services are formalized by HAH and patients are transferred to HAH after a medical prescription. The presence of at least one caregiver at home and the participation of the general practitioner are recommended before patients come back home but are not mandatory.

The study has been lead in Greater Paris University Hospital’s HAH named “AP-HP’s HAH” (Assistance Publique - Hôpitaux de Paris). AP-HP’s HAH is organized through the largest conglomerate of 37 public hospitals located in Paris and suburbs, seeing 7 millions of patients yearly and considered a separate hospital within that conglomerate. It is the first HAH institution implemented in France in 1957 and the biggest public one with approximately 800 patients/day. The institution covers all Paris plus three departments (Hauts-de-Seine, Seine Saint Denis, Val de Marne) and has 20 territorial bases co-located in existing hospitals. Aside from adult medicine, AP-HP’s HAH has specialized obstetric and pediatric services, and a dedicated after-hours service.

### Study population

The study population was recruited in 2017 in one care unit from AP-HP’s HAH, covering the 9th, 17th and 18th districts of Paris. These three districts are representative of the Parisian population.

All patients and caregivers in the unit between March and May 2017 were recruited according to the following criteria. The inclusion criteria were: patients older than 16 years old speaking fluently French, physically and mentally able to answer questions, with a vital prognosis superior to three months, and a family caregiver. The exclusion criteria were: the lack of consent from the patient or the caregiver, the interruption of the HAH process between the inclusion and the interview, a stay in HAH shorter than a week, or short and repetitive stays, the variation of caregivers.

The duos of patients and caregivers were chosen among the ongoing files of patients followed in this HAH care unit. The nurse supervisor was given the inclusion and exclusion criteria. She highlighted every matching file during the whole process of selection, and then submitted them to the research team (formed by three physicians, one being a public health medical resident, one a gerontologist with a double qualification on public health and the last being a public health doctor) for a verification of the match. After this step, the nurse supervisor made the first contact with every patient and caregiver to explain the goal of the study, the protocol, and ask them for an appointment with the public health resident in charge of the study. During the appointment, signed consent was obtained from both patient and caregiver, after due information. One patient/caregiver duo was not included due to the unwillingness to participate of the caregiver. One duo was considered for participation but the patient died suddenly before the inclusion process was over. No differences were noted between patient’s profiles, respondents and non-respondents. The inclusion process was stopped after data saturation was reached. Nineteen interviews were realized with nine patients and ten caregivers.

Among the nine patients, five were men and four women, with an average age of 68. Their main pathologies that got them admitted in HAH, were very different from one another: 56% of patients were admitted for a cancer, 22% for a chronic disease (such as diabetes) and 22% for post-fracture care.

Among the caregivers, six were men and four women, with an average age of 57. Seven were the patient’s spouse/partner, two were the patient’s child and one was the patient’s son-in-law. 50% of the caregivers had a professional activity (Table 1).

**Table 1:** Social characteristics of caregivers

Caregivers	Age	Cohabitation	Family status
C1	35–45	Yes	Daughter
C2	35–45	Yes	Son-in-law
C3	65–75	Yes	Husband
C4	65–75	Yes	Wife
C5	65–75	Yes	Husband
C6	35–45	No	Son
C7	55–65	Yes	Wife
C8	55–65	Yes	Wife
C9	65–75	Yes	Husband
C10	55–65	Yes	Husband

The study was approved by the Hospital at home review board and by the HAH ethical committee. Participants received a written information file describing the design and modalities of the study and then, after being given the opportunity to ask questions if they wished to, written consent was obtained from all of them.

### Data collection

Qualitative data came from semi-directed separate face-to-face interviews of both caregivers and patients. The same person, a public health resident, trained in qualitative research and one of the study’s investigators, conducted all the interviews. They were held in each patient’s and caregiver’s home, except once, when the caregiver wanted to meet away from her home, and lasted from 15 to 115 min.

The design of the questionnaire was based on a review of the literature and was validated along with the protocol by a group of HAH professionals volunteers during a weekly staff meeting. This reunion included a dozen of HAH professionals (doctors, nurses, assistant-nurses, psychologist). Their feedback mainly concerned the need to not only target patients ongoing supportive care but also suffering from a wider spectrum of diseases, and to take into account every step of HAH (from hospital release to HAH discharge).

One test session of interviews was realized in order to validate the content of the questionnaire and the process. One interview of a patient and one of each of his two caregivers were realized during this session. The data from the test interviews was included in the final data set.

All the interviews began with a very general question concerning both patient’s and caregiver’s current quality of life and then moved on to more specific questions exploring four main themes: 1) their participation to the decision of transfer to HAH and motivation(s) 2) their opinions on the advantages and barriers of home-care provided by AP-HP’s HAH (including questions on quality, simplicity, coordination, reactivity, self-participation and relationships with healthcare professionals), 3) the presence of an impact on various health parameters since the transfer of the patients and 4) the relationship between caregivers and patients during the time of the care and its evolvement. The questionnaire and interview guide used in the study were developed for this study and have not been published elsewhere (Additional file 1).

Having caregivers fill in the Zarit Burden Interview (ZBI) after their face-to-face interview, in another room, separated from the patient, and collected quantitative data. We used the revised version of the ZBI with 22 items to evaluate the psychological condition of the caregivers [34]. 12 items or the 29 items versions were also available. But although the 29 items version has an excellent internal consistency, we, as the majority of researchers nowadays, judged it too long. The shorter, 12 items version was, this time, not complete enough. The 22-items version of the ZBI was close enough of our questionnaire’s themes to be of interest.

Each item on the interview is a statement, which the caregiver is asked to endorse, using a 5-points scale. Response options range from 0 (Never) to 4 (Nearly Always).

The obtained score may vary from 0 to 88, and the results are categorized in “little or no burden” (0–21), “mild to moderate burden” (21–40), “moderate to severe burden” (41–60), “severe burden” (> 60).

### Coding and analysis

Transcripts were produced, read and coded by the three researchers. The coding and analysis were performed using the Strauss and Corbin grounded theory approach, consisting of an open, axial and selective coding [29] in order to identify relevant categories and relationships. Following the rules of axial coding strategy [29], codes with the same meaning were grouped into categories (e.g. organization problems). Then the identified categories were further developed, refined and brought together to describe a bigger picture. (e.g. the impact of HAH on the caregiver). The analytical process was repeated until saturation (the point at which additional analysis only repeats previous interpretation). A preliminary categorization of findings took place following the first 11 interviews (5 patients and 6 caregivers), saturation emerged after 15 persons (7 patients and 8 caregivers) were interviewed and the remaining data was collected and used to validate the categories. The researchers compared their own analysis and discussed the final coding scheme. This separation of reflections, within the early stages of the analysis, not only enabled a clear personal overview of the topic and of the categories, but also the practical application of the concept of “collective intelligence”, that is the ability of a group to outperform individuals in solving cognitive tasks.

## Results

Findings tend to identify the point of view of family caregivers’ (C) and patients’ (P) relatives about care during the hospitalization at home.

### Qualitative results

#### A lack of knowledge regarding the existence of HAH, of information throughout the whole implementation process but a positive predictive outlook of HAH both from patients and caregivers

None of the patients or caregivers (except two of them, working in the healthcare system) knew the existence of HAH before it was presented to them.

*“I didn’t know HAH existed”* P4

*“A huge discovery! We didn’t know it!”* C2

For some of them, the admission in HAH was perceived as an obligation, mostly because there was no alternative or because the only other solution was an admission in an institution very far from their home.

*“The social worker, as they couldn’t keep him, asked us - even more or less convoked us - to find a solution. Basically, we had no other choice than HAH. It really wasn’t our first choice. We wanted him to go to a rehabilitation center so that he could recover. Especially for his leg, so he could walk again. For us, it really has been imposed. They couldn’t keep him longer and they couldn’t find a place in a rehabilitation facility.”* C1

Recurring complaint of the caregivers, the lack of a precise and realistic information on the practical functioning of HAH, before the decision of admission is made, resulted in some of the caregivers not realizing how important and absorbing HAH can be, and ending up lost, disappointed or with a feeling of having been deluded.

*“HAH was sold to us as “hospital at home”. The same as a real hospital. I did really think it would be the same. But no. It is not the same.”* C1

Nevertheless, in any case, HAH’s implementation has been perceived as a positive event.

“*We had no hesitation on HAH. It was clear that the most satisfying solution was him coming back home. ”* C1

#### The versatility of caregivers’ role leads to much suffering but diverse sources of support exist or are implemented

Caregivers take care of administrative and daily stewardship, but also manage medical and material emergencies. They also have to handle the coordination between hospitals and HAH teams and are a very strong moral support for the patient. Despite very variable situations, every caregiver described the same basic tasks/chores (Table 2).

*“I’m a multi-functional maid. Almost madam’s slave.”* C9

*“(The role of the caregiver is) The stewardship. All the stewardship. And from time to time also the driver”* P3

*“And in my father’s case, when he suffers from complications, at the end I always have to take him back to the hospital, nothing can be done at home, there is no solution, every time, we have to go to the hospital.”* C1

Caregivers suffer from a multifaceted exhaustion, both physical and mental. They all are stressed out and sometimes close to a burnout. The toughest situations concern the ones working full-time beside their role of caregiver.

“*My wife broke down just yesterday, because it starts to become very difficult to handle. He’s been out of the hospital for 6 months now, she is really tired, because of her job, and she has to take care of her father, cook, deal with daily issues, with tiredness…”* C2

Caregivers also need moral support. Some already have a very strong support system (family/friends), other don’t. Loneliness is very tough in those situations.

*“We preserve the family unit. It helps the patient”* C6

Both patients and caregivers often rely on HAH staff, considered as being way closer to them than when being at the hospital.

*“They are here to lift up our mood a little… Talk... They listen... As it isn’t always easy, it feels good to have this staff… We know them, we talk… It enables us to vent when things are not going well…”* C1

Religion is also an important resource of relief, or support. Most of the interviewed persons highlighted the fact that they rely very much on religious beliefs, but also practice (prayers, visits of religious edifices….). Three religions were mainly represented among them (catholic, jewish and muslim religions). Some, however, refused to talk about this topic, and some declared themselves atheists.

“*Religion helped me, yes, it really helped me. I am muslim”* C1

**Table 2:** Main daily tasks of caregivers (extracted from verbatim)

Caregivers	Main daily tasks
C1	Clinical monitoring of the patient night and days (and of the technical equipment) Change of work schedule to help the patient (very early in the morning, alternating work with C2 so someone is always present) Help to move the patient (Carrying and accompanying the patient: toilets, shower..) Replacement of dirty sheets Cooking for the patient Accompaniment of the patient outside and to the hospital Daily organization Management of medical appointments Coordination between hospital and HAH Management of medical emergencies (coordination between HAH/ambulances and accompaniment to the ER) Management of the paperwork Moral support
C2	Clinical monitoring of the patient night and days (and of the technical equipment) Change of work schedule to help the patient (very late in the evening, alternating with C1 so someone is always present) Help to move the patient (Carrying and accompanying the patient: toilets, shower..) Accompaniment of the patient outside and to the hospital Daily organization Management of medical emergencies (coordination with HAH/ambulances and accompaniment to the ER) Moral support
C3	Management of the paperwork Coordination with the hospital, management of the medical appointment and of the transfers to the hospital and back Accompaniment of the patient outside Daily organization Takes part to the nursing care Carrying the patient (toilets, shower) Moral support
C4	Accompaniment of the patient to the hospital Preparation of the daily equipment used for nursing care Clinical monitoring Moral support
C5	Sleeps on the couch because of the medical bed Cleaning of the house Accompaniment of the patient to the hospital Carrying of the patient Presence and participation during the nursing care Moral support
C6	Participation to the patient’s hygiene Participation to the nursing care Daily preparation of the pillbox Coordination with the hospital (appointments, prescriptions) and between the hospital and HAH Management of the patient’s transfers to the hospital and back Management of the meals Moral support
C7	Management of medical emergencies (coordination with HAH/ambulances and accompaniment to the ER) Carrying the patient Drives the patient to appointments Participation to nursing care Daily preparation of the medications Moral support
C8	Management of technical emergencies Participation to nursing care Management of daily organization Change of work schedule to help the patient (comes back to prepare lunch and stopped all external activities to be home) Clinical monitoring of the patient and equipment Moral support
C9	Daily shopping and meal preparation Accompaniment of the patient to the hospital Moral support
C10	Daily organization Accompaniment of the patient to the hospital Moral support

*“ Religion helps me a lot. I am jewish and proud of it. It helps me being structured. (…) And the more my husband was sick, the more religious he got. (…) I think his religious side prevents him from being kind of suicidal.” C4*

#### HAH is linked to an improvement of morale and a good quality of care but necessitates the integration of heavy organizational constraints by caregivers and patients

Both patients and caregivers were satisfied with the return home and declared that their morale is much improved. Being at home also increased the patients’ appetite (hospital food being heavily criticized).

*“We eat a hundred times better at home than in the hospital”* P7

*“My morale is better too.”* P4

*“ (Is it better for him to be treated at home?) Yes, for his morale.”* C1

They are also pleased with the quality of care.

*“There always is somebody who listens, who fastly comes if there is a problem… It’s an « à la carte » service.”* P9

*“We can heavily insist on the fact that every person who comes is remarkably competent and nice. And we admire those people who work hard, believe in what they are doing and are not looking to have a career.”* C3

However the care schedule’s high variability, a major staff turnover and the obligation of home-storage of medical material were described as real constraints. A family had to move into another apartment because of the space the material was taking, and one of the caregivers has to sleep on the couch, because the single medical bed filled the whole bedroom.

*“We never were quiet, especially as there never was a permanent schedule. It is not easy. It is completely random. Some nurses prefer to start the day with my dad, other to finish by him. It depends.”* C1

*“Everyday, there are different people. It is disturbing.”* C6

*“I won’t lie to you; we start to walk on each other’s feet. With all this material, the wheelchair, the commode chair, the patient lift, the medical bed... There was no space left! I had to ask for a bigger apartment and luckily I got this one.”* C2

#### Home care strongly impacts the patient/caregiver relationship, but so does the disease itself

The whole process of at-home care can harm the relationship between the patient and his caregiver: fatigue, organizational constraints, stress… All of those are factors of harm in a relationship.

But sometimes the hardships reinforce the relationship.

*“Sometimes we fight. He became more capricious.”* C1

*“He is more thoughtful, he cares more, I think. Maybe he sometimes thinks I don’t take it enough upon myself.”* P2

The vast majority of patients were aware of the difficulties faced by their caregiver and felt guilty about it, which lead to suffering. They considered themselves a “burden” or a “weight”.

*“I can feel I am a weight for her. I think I am a very heavy weight for her.”* P1

*“ It might even be harder for him than it is for me, you see? To bear my mood swings, I think it is hard. I know I am sick, it is my problem. But for him it is difficult. It feels that way.”* P2

Another source of suffering for the patient and the caregiver were the intrusion of the staff in their intimacy, but also the forced intimacy that was created when a child had to take care of one of his parents and had, for example, to see them naked, during care or when helping his parent to go to the bathroom.

*“There is a great modesty in our family. It was a humiliation for her. The relationship with the body is very different in the hospital. With the family, it’s disturbing because it is humiliating for my mother, because she really is very modest and to see her son witness her in the middle of her excrements on the floor… It was horrible.”* C6

*“The intimacy is touched, hurt, disturbed... When I felt that lots of people were coming, whenever the time, you could never know when, or who was going to come. ”* C5

Finally, the existence of the disease is in itself a huge factor of change in their relationship. Caregivers worry and suffer from the sight of their suffering sick relative. This, unfortunately, is the only element on which no one has control over.

### Quantitative results

The mean score was 22,4/88 (9–47), meaning that the burden score is “very low”.

The three questions with the highest scores were: “Do you feel your relative is dependent on you?” (total score of 24/40), then “Do you feel that your relative seems to expect you to take care of him/her as if you seem the only one he/she could depend on?” (total score of 21/40) and “Are you afraid what the future holds for your relative?” (total score of 19/40).

The questions with the lowest score were: “Do you feel that you don’t have enough money to take care of your relative in addition to the rest of your expenses?” (2/40), then “Do you feel uncertain about what to do about your relative?” (4/40) and “Do you feel you have lost control of your life since your relative’s illness?” (4/40).

Caregivers who are working have a mean score of 26/40 meanwhile those who aren’t, have a mean score of 19,6/40. The detailed answers to the 22 questions are in the Additional file 2.

The mean score was 22,4/88 (9–47), meaning that the burden score is “very low”.

## Discussion

We found that HAH remains widely unknown among patients and caregivers, who rarely are at the origin of the admission, and lack information before the return home. Then we understood the role of the caregivers and its versatility, their suffering but also their sources of comfort during the stay in HAH. Moreover, we analyzed that the return home was considered satisfying thanks to a good quality of care and an improvement of the health parameters despite imposing a real organizational constraint. Finally, the impact of HAH on the patient/caregiver relationship was important and related both to the disease (and its consequences) and to the home care. Data from the ZBI study showed an underestimation of the caregiver’s own burden.

Our study enlightened the fact that patients and caregivers weren’t aware of the existence of HAH. Access to healthcare is a fundamental right according to the European Union’s fundamental rights charter and the horizontal equity principle, which states that the treatment must be equal to the need [35]. But studies show that the level of inequalities in healthcare access significantly varies from one country to another. They observe the same inequalities of use of specialized care in some EU countries - despite a universal health coverage principle - and informational barriers are considered a key element [36, 37]. There are thus still informational issues that need to be addressed in the French healthcare system. Health care users have to be better informed about the range of health care services available in their community. Each patient should be in capacity to choose where he (she) wants to be taken care of, may it be in a conventional hospital or in HAH. The development of HAH has to include its promotion towards care users and healthcare professionals, in addition to a reinforcement of its bonds with hospitals.

The role of the family caregiver in HAH was huge with multi-tasks interventions, including instrumental and basic activities of daily living and care coordination. Our results highlighted the complex role of family caregivers, these family members who help but also often provide care. HAH services are available 24/7 but no healthcare professional is staying all the time. That is mostly why caregivers were solicited to help and participate to the continuity of care, within a multidisciplinary team. Given the intensity and frequency of the care, this system implies that caregivers are supposed to often be involved. Even if the technical care was provided by the HAH team, caregivers were associated, and also dealing with the logistic tasks. Caregivers appeared as the daily witnesses for HAH professionals of the evolutions of the patient’s health, and mostly were the link between HAH and the hospital, despite the obvious fact that they should not be. Moreover, if the health consequences for the caregivers have been demonstrated on physical and mental dimensions [38–40], our findings showed that the degree of burden was divided. Results of the ZBI were very low and contrasted with what caregivers said during the interviews. The primary idea of asking the caregivers to fill in the ZBI was to be able to adjust the weight of their words. If after having raised an issue during the interview, the caregiver confirmed by picking an elevated score to the related ZBI question, it insists on the importance of the problem. But we had the surprise to find some Zarit answers completely opposed to what had been said orally. Sagne and al. describe the same phenomenon but explain it by an improvement of the caregiver’s mood after having let off the steam during the interview [41]. We, however, think that caregivers tend to severely under-estimate their suffering and some other studies tend to approve this hypothesis [42]. The written form, less spontaneous, can lead to the caregiver thinking twice. They might then think they exaggerated when previously giving their opinion. It might also be very complicated for caregivers, especially when they work full-time, to admit to themselves, when they face that very harsh questionnaire, that taking care of their loved one can be tough and cause them pain.

If the perceptions of caregivers were mitigated, the patients seemed to be more satisfied to come back home. A recent Cochrane review on hospital at home services admits that patients who receive care at home may be more satisfied than those who are in hospitals [43]. However, another Cochrane study explains that concerning functionally dependent older patients, there is insufficient high-quality published data to support any particular model of care and that future studies should take into account caregivers’ burden besides performing economic analysis and assessing healthcare utilization [44].

Our findings enabled us to better understand the impact of HAH on the patient/caregiver relationship. Patients felt guilty of their caregiver’s suffering, which thus caused them pain too. Caregivers felt sad because of the patient’s sickness and daily pain, but they also had to face the patient’s mood changes, and saw their own workload strongly increase because of the home care, which could lead to a real deterioration of their relationship, but also of the caregiver’s health. Our work brought up a fundamental question: can we, ethically, favor patient’s well-being over caregiver’s suffering? If HAH is beneficial to patients but strongly impacts caregivers, should we deprive the patient from a better care to relieve the caregiver? Or should we force the caregiver to bear the situation in the name of “good care”? If nothing is made to smoothen out caregivers’ life conditions, this question, which is still luckily mainly rhetorical, could become a real ethical dilemma in the upcoming years. Yet the impact of being a caregiver is very well documented for certain diseases (38) and both policy makers and associations are slowly starting to take actions. However, a more general approach of the information, the formation and the support of caregivers needs to be implemented in parallel of the development of HAH.

We should acknowledge some of the limitations of this study. The sample of patients and caregivers came from the city of Paris where care stakeholders are numerous and fragmented between hospitals and community care, making the coordination of care more difficult for HAH. Moreover, given the fact that neither patients nor caregivers had initially chosen to come back home with the HAH, the sample was composed of participants who did not know how HAH usually unfolds and it might have had an impact on the answers and the reactions. We also have not studied the patients and caregivers who were included in the HAH system from a long-term care sector for elderly dependent person with cognitive impairment. We haven’t interviewed care professionals from HAH and their perceptions could have brought up complementary information about the care in HAH. We did not ask for the city of origin of patients and caregivers, while cultural specifics can be found in different regions or countries and impact the behavior. Finally, the sample was small to sufficiently support the quantitative analysis and to take into account the diversity of situations. However, the early saturation of data, especially concerning caregivers’ situation, highlights that despite very different situations, the issues faced by caregivers remain the same. It strongly encourages us to dig deeper on this topic, and shows the need of other studies. But even with these limitations, this study remains the first to take into account patients’ and caregivers’ perceptions about complex care at home.

This work enabled the highlight of - until now- ignored issues that will need to be deepened in further studies.

HAH strongly involves caregivers all along the process and they are the keystone of this system. HAH development necessitates to associate patient and caregivers to decisions and to take into account their needs and their relationship, at every step. Otherwise, this study highlights the need to better assess the ability of the caregiver to cope with his or her relative in the context of HAH with acute and subacute care at home. Public policies push for a faster expansion of HAH in France but also all across the industrialized world. Accelerating its development without questioning its current functioning means forcing more and more caregivers to carry the huge weight of this system.

## Additional files

Additional file 1: Zarit burden inventory’s answers from the caregivers’ population. (DOCX 17 kb)

Additional file 2: Zarit burden inventory’s results. (DOCX 22 kb)

## Data Availability

The datasets used and analyzed during the current study are available from the corresponding author upon request.

## Abbreviations

APHP: Assistance Publique - Hôpitaux de Paris
HAH: Hospital-At-Home

## References

[1] Caplan GA, Sulaiman NS, Mangin DA, Aimonino Ricauda N, Wilson AD, Barclay L (2012). A meta-analysis of "hospital in the home". Med J Aust..

[2] Caplan GA (2008). Does ‘Hospital in the Home’ treatment prevent delirium?. Aging Health.

[3] Leff B, Burton L, Mader SL (2005). Hospital at home: feasibility and outcomes of a program to provide hospital-level care at home for acutely ill older patients. Ann Intern Med.

[4] Stessman J, Ginsberg G, Hammerman-Rozenberg R (1996). Decreased hospital utilization by older adults attributable to a home hospitalization program. J Am Geriatr Soc.

[5] Langhorne P, Taylor G, Murray G (2005). Early supported discharge for stroke patients: a meta-analysis of individual patients’ data. Lancet.

[6] Messecar D. (2009). Review: admission-avoidance hospital-at-home decreases mortality at 6 months but does not differ from inpatient care for readmission. Evidence-Based Nursing.

[7] Shepperd S, Doll H, Broad J, et al. Early discharge hospital at home. Cochrane Database Syst Rev. 2009;(1):CD000356. 10.1002/14651858.CD000356.pub3. 10.1002/14651858.CD000356.pub3 PMC4175532 19160179

[8] Leff B, Montalto M (2004). Home hospital -- toward a tighter definition. J Am Geriatr Soc.

[9] Caplan GA, Brown A. Post acute care: can hospitals do better with less? Aust Health Rev 1997; 20: 43–54. Availability: http://search.informit.com.au/documentSummary;dn=971111718;res=IELAPA. 10.1071/ah970043a 10169367

[10] Wells M, Harrow A, Donnan P (2004). Patient, carer and health service outcomes of nurse-led early discharge after breast cancer surgery: a randomised controlled trial. Br J Cancer.

[11] Siggeirsdottir K, Olafsson O, Jonsson H (2005). Short hospital stay augmented with education and home-based rehabilitation improves function and quality of life after hip replacement: randomized study of 50 patients with 6 months of follow-up. Acta Orthop.

[12] Aujesky D, Roy PM, Verschuren F (2011). Outpatient versus inpatient treatment for patients with acute pulmonary embolism: an international, open-label, randomised, non-inferiority trial. Lancet.

[13] Mendoza H, Martin MJ, García A (2009). ‘Hospital at home’ care model as an effective alternative in the management of decompensated chronic heart failure. Eur J Heart Fail.

[14] Caplan GA, Coconis J, Board N (2006). Does home treatment affect delirium? A randomised controlled trial of rehabilitation of elderly and Care at Home Or Usual Treatment (the REACH-OUT trial). Age Ageing.

[15] TIBALDI V., AIMONINO N., PONZETTO M., STASI M.F., AMATI D., RASPO S., ROGLIA D., MOLASCHI M., FABRIS F. (2004). A RANDOMIZED CONTROLLED TRIAL OF A HOME HOSPITAL INTERVENTION FOR FRAIL ELDERLY DEMENTED PATIENTS: BEHAVIORAL DISTURBANCES AND CAREGIVER’S STRESS. Archives of Gerontology and Geriatrics.

[16] Aimonino Ricauda N, Tibaldi V, Leff B (2008). Substitutive “hospital at home” versus inpatient care for elderly patients with exacerbations of chronic obstructive pulmonary disease: a prospective randomized, controlled trial. J Am Geriatr Soc.

[17] Carratalà J, Fernández-Sabé N, Ortega L (2005). Outpatient care compared with hospitalization for community-acquired pneumonia: a randomized trial in lowrisk patients. Ann Intern Med.

[18] Corwin P, Toop L, McGeoch G (2005). Randomised controlled trial of intravenous antibiotic treatment for cellulitis at home compared with hospital. BMJ.

[19] Ministère de la Santé. https://solidarites-sante.gouv.fr/soins-et-maladies/prises-en-charge-specialisees/had-10951/had

[20] Emanuel E, Fairclough D, Slutsman J, Alpert H, Baldwin D, Emanuel L (1999). Assistance from family members, friends, paid care givers, and volunteers in the care of terminally ill patients. N Engl J Med.

[21] Buyck JF, Bonnaud S, Boumendil A, Andrieu S, Bonenfant S, Goldberg M, Zins M, Ankri J (2011). Informal caregiving and self-reported mental and physical health:results from the Gazel cohort study. Am J Public Health.

[22] Gaugler JE, Yu F, Krichbaum K, Wyman JF (2009). Predictors of nursing home admission for persons with dementia. Med Care.

[23] Michalowsky Bernhard, Flessa Steffen, Eichler Tilly, Hertel Johannes, Dreier Adina, Zwingmann Ina, Wucherer Diana, Rau Henriette, Thyrian Jochen René, Hoffmann Wolfgang (2017). Healthcare utilization and costs in primary care patients with dementia: baseline results of the DelpHi-trial. The European Journal of Health Economics.

[24] Vitaliano PP, Schulz R, Kiecolt-Glaser J, Grant I (1997). Research on physiological and physical concomitants of caregiving: where do we go from here?. Ann Behav Med.

[25] Carretero S, Garcés J, Ródenas F, Sanjosé V (2009). The informal caregiver’s burden of dependent people: theory and empirical review. Arch Gerontol Geriatr.

[26] Pinquart M, Sörensen S (2003). Differences between caregivers and noncaregivers in psychological health and physical health: a meta-analysis. Psychol Aging.

[27] de Vugt ME, Jolles J, van Osch L (2006). Cognitive functioning in spousal caregivers of dementia patients: findings from the prospective MAASBED study. Age Ageing.

[28] Mackenzie CS, Wiprzycka UJ, Hasher L, Goldstein D (2009). Associations between psychological distress, learning, and memory in spouse caregivers of older adults. J Gerontol B Psychol Sci Soc Sci.

[29] Strauss A. Basics of qualitative research : techniques and procedures for developing theory. 2nd edition. Thousand Oaks. Sage. 1998.

[30] Décret no 92–1101 du 2 octobre 1992 relatif aux structures de soins alternatives à l'hospitalisation mentionnées à l'article L. 712–2 du code de la santé publique (1992). Availability: https://www.legifrance.gouv.fr/affichTexte.do?cidTexte=JORFTEXT000000726288&categorieLien=id.

[31] Loi n° 2009–879 du 21 juillet 2009 portant sur la réforme de l'hôpital et relative aux patients, à la santé et aux territoires (2009). Availability: https://www.legifrance.gouv.fr/affichTexte.do?cidTexte=JORFTEXT000020879475&categorieLien=id.

[32] Rapport d'activité 2014-2015. Fédération Nationale des Etablissements d'Hospitalisation A Domicile (FNEHAD) 2015. Availability: https://www.fnehad.fr/?s=rapport+activit%C3%A9.

[33] Ministère de la santé et des Solidarités. Circulaire n° DHOS/O3/2006/506 du 1er décembre 2006 relative à l’hospitalisation à domicile (HAD). Paris; 2006. Availability: circulaires.legifrance.gouv.fr/pdf/2009/04/cir_7220.pdf.

[34] Zarit SH, Reever KE, Bach-Peterson J (1980). Relatives of the impaired elderly: correlates of feelings of burden. Gerontologist..

[35] Or Z, Jusot F, Yilmaz E (2009). Inégalités de recours aux soins en Europe: Quel rôle attribuable aux systèmes de santé ?. Revue économique.

[36] Grossman M. The human capital model. In: Dans Culyer AJ, Newhouse JP, editors. Handbooks of health economics, vol. 1 a. Amsterdam: Elsevier (North-Holland); 2000. p. 348–408.

[37] Campbell D, Braden O, Gibson K, Thurston W. Primary healthcare needs and barriers to care among Calgary’s homeless populations. BMC Fam Pract. 2015;16(139). 10.1186/s12875-015-0361-3. 10.1186/s12875-015-0361-3 PMC4603688 26463577

[38] Oberst M, Thomas S, Gass K, Ward S (1989). Caregiving demands and appraisal of stress among family caregivers. Cancer Nurs.

[39] McMillan S, Mahon M (1994). The impact of hospice services on the quality of life of primary caregivers. Oncol Nurs Forum.

[40] Roepke S, Mausbach B, Patterson T, von Känel R (2011). Effect of Alzeihmer caregiving on allostatic load. J Health Psychol.

[41] Sagne A., Girtanner Ch., Blanc P., Duboeuf G., Gonthier R. (2004). Evaluation par l’échelle de Zarit d’une prise en charge psychologique des aidants de patients atteints de syndrome démentiel. NPG Neurologie - Psychiatrie - Gériatrie.

[42] Antoine P, Quandalle S, Christophe V. Vivre avec un proche malade: évaluation des dimensions positives et négatives de l’expérience des aidants naturels. Ann Méd Psychol. 2010;168(4):273–82. Available: https://hal.archives-ouvertes.fr/hal-00638570/document.

[43] Shepperd S, Iliffe S, Doll H. Admission avoidance hospital at home. Cochrane Database systematic Review. 2016. 10.1002/14651858.CD007491. 10.1002/14651858.CD007491.pub2 PMC6457791 27583824

[44] Young C, Hall A, Gonçalves-Bradley D, Quin T, Hooft L, Munster B, et al. Home or foster home care versus institutional long-term care for functionally dependent older people. Cochrane Database Syst Rev. 2017. 10.1002/14651858.CD007491.pub2. 10.1002/14651858.CD009844.pub2 PMC6478250 28368550

