# Supplementary material for: The experience of patients and family caregivers during hospital-at-home in France
Source: BMC Health Serv Res. 2019 Jul 9;19:470. doi: 10.1186/s12913-019-4295-7 (PMC6617632; doi:10.1186/s12913-019-4295-7)
Supplement: Supplementary file 1 — Zarit burden inventory’s answers from the caregivers’ population. (DOCX 17 kb) [file 12913_2019_4295_MOESM1_ESM.docx]

Additional file 1: Questionnaire and interview guide for caregivers and patients

Interview guide for caregivers

Introduction

Hello. My name is H.R. and I am a public health medical resident in Greater Paris University Hospitals’ HAH. First, I’d like to thank you to have agreed to meet me. As you are aware of it, I currently am conducting a study on both family caregivers’ and patients’ experience throughout an HAH stay.

For this purpose, I’d like to interview you. You are free to participate or not to the study.

If you agree, I will then record the interview to ensure the accuracy of the data. The analyze of our conversation will then be anonymized in a way participants can’t be identified, or published direct comments can’t be linked to them.

About HAH

- How did you enter the HAH system?

What is your own request? The patient’s? A proposition from the health care team? Where was the patient before HAH?

- If the request was yours, did the patient immediately agree or did he/she have to be convinced? If so, why?
- According to you, what are the pros and cons of HAH?

In terms of care coordination? (Time of arrival of health care professionals to the patient’s home? Frequency of the professionals turn over? Easiness of the process?)
What is your perception of the tasks you have to do by yourself?

Is there a financial impact for you? An impact on your work?

Does HAH bring something more than the previous type of care the patient was undergoing?

Do you know any other patient in HAH right now? If so, did it help you in your choice to pick HAH?

Feelings about the situation

- Has someone close to you died? If so, was it in HAH?
- How do you perceive and feel about the changes brought by HAH in your home?
  (medicalized bed, stock of products, oxygen bottles….)
- What is helping you hold on?

Religion? Social support system (family/friends)? Support group? Associations?

About the patient

- Has HAH changed your relationship with the patient? If so, in what way?

Has the patient changed his/her behavior since the return-home?

How do you think the patient feels about HAH?

How do you think the patient thinks about the way you feel about HAH?

Zarit Burden Interview

Please, fill in the questionnaire. Is there any other information you want to give us or any important topic you feel might be relevant?

About the caregiver:

Gender

Type of relationship with the patient (parent, child, spouse...)

Do the caregiver and the patient share their home?

Interview guide for patients

Introduction

Hello. My name is H. R. and I am a public health medical resident in Greater Paris University Hospitals’ HAH. First, I’d like to thank you to have agreed to meet me. As you are aware of it, I currently am conducting a study on both family caregivers’ and patients’ experience throughout an HAH stay.

For this purpose, I’d like to interview you. You are free to participate or not to the study.

If you agree, I will then record the interview to ensure the accuracy of the data. The analyze of our conversation will then be anonymized in a way participants can’t be identified, or published direct comments can’t be linked to them.

About HAH

- How did you enter the HAH system?

What is your own request? Your caregiver’s? A proposition from the health care team? Where were you taken care of before HAH?

- If the request was yours, did your family immediately agree or did they have to be convinced? If so, why?
- According to you, what are the pros and cons of HAH?

In terms of care coordination? (Time of arrival of health care professionals to the patient’s home? Frequency of the professionals turn over? Easiness of the process?)

Feelings about the situation

- How do you feel since your return-home?

Do you have more appetite? If you have pets, was it important for you to be reunited with them?

If you smoke/drink, was it important for you to be able to do it?

Are there any other factors impacting the way you feel about your return-home in HAH?

About your caregiver

- Has HAH changed your relationship with your caregiver?

How do you feel about this very strong intimacy created by HAH?

Has your caregiver changed his behavior since the beginning of HAH?

How do you think he/she feels about this whole situation?

How do you think he/she thinks about how you feel about this whole situation?

Thank you very much. Is there any other information you want to give us or any important topic you feel might be relevant?
