# Supplementary material for: The experience of patients and family caregivers during hospital-at-home in France
Source: BMC Health Serv Res. 2019 Jul 9;19:470. doi: 10.1186/s12913-019-4295-7 (PMC6617632; doi:10.1186/s12913-019-4295-7)
Supplement: Supplementary file 2 — Zarit burden inventory’s results. (DOCX 22 kb) [file 12913_2019_4295_MOESM2_ESM.docx]

Additional file 2: Zarit burden inventory’s results

|  | C1 | C2 | C3 | C4 | C5 | C6 | C7 | C8 | C9 | C1 | Total |
| --- | --- | --- | --- | --- | --- | --- | --- | --- | --- | --- | --- |
| Do you feel that your relative asks for more help than he/she needs? | 2 | 2 | 0 | 2 | 2 | 0 | 2 | 1 | 1 | 0 | 12 |
| Do you feel that because of the time you spend with your relative you don’t have enough time for yourself? | 2 | 3 | 1 | 0 | 1 | 2 | 2 | 3 | 2 | 0 | 16 |
| Do you feel stressed between caring for your relative and trying to meet other responsibilities for your family or work? | 2 | 3 | 0 | 0 | 3 | 2 | 2 | 3 | 1 | 0 | 16 |
| Do you feel embarrassed over your relative’s behavior? | 1 | 2 | 0 | 1 | 1 | 0 | 0 | 0 | 3 | 0 | 8 |
| Do you feel angry when you are around your relative? | 0 | 1 | 0 | 1 | 1 | 2 | 2 | 0 | 1 | 0 | 8 |
| Do you feel that your relative currently affects your relationships with other family members or friends in a negative way? | 0 | 2 | 0 | 0 | 0 | 0 | 1 | 0 | 1 | 0 | 4 |
| Are you afraid of what the future holds for your relative? | 4 | 1 | 2 | 3 | 2 | 3 | 3 | 0 | 1 | 0 | 19 |
| Do you feel your relative is dependent on you? | 2 | 4 | 1 | 3 | 3 | 3 | 3 | 0 | 1 | 4 | 24 |
| Do you feel strained when you are around your relative? | 0 | 0 | 0 | 0 | 1 | 2 | 1 | 0 | 2 | 0 | 6 |
| Do you feel your health has suffered because of your involvement with your relative? | 2 | 0 | 2 | 0 | 2 | 0 | 1 | 0 | 0 | 0 | 7 |
| Do you feel like you don’t have as much privacy as you would like because of your relative? | 2 | 4 | 0 | 2 | 0 | 1 | 0 | 0 | 1 | 0 | 10 |
| Do you feel that your social life has suffered because you are caring for your relative? | 3 | 3 | 1 | 0 | 1 | 0 | 1 | 0 | 2 | 0 | 11 |
| Do you feel uncomfortable about having friends over because of relative? | 0 | 4 | 0 | 2 | 0 | 0 | 0 | 3 | 1 | 0 | 10 |
| Do you feel that your relative seems to expect you to take care of him/her as if you were the only one he/she could depend on? | 2 | 4 | 0 | 3 | 1 | 1 | 3 | 1 | 3 | 3 | 21 |
| Do you feel that you don’t have enough money to take care of your relative in addition to the rest of your expenses? | 0 | 2 | 0 | 0 | 0 | 0 | 0 | 0 | 0 | 0 | 2 |
| Do you feel that you will be unable to take care of your relative much longer? | 1 | 2 | 1 | 0 | 0 | 0 | 0 | 0 | 3 | 0 | 7 |
| Do you feel that you have lost control of your life since your relative’s illness? | 0 | 0 | 1 | 0 | 0 | 2 | 0 | 0 | 1 | 0 | 4 |
| Do you wish you could leave the care of your relative to someone else? | 1 | 3 | 0 | 0 | 2 | 3 | 1 | 0 | 1 | 0 | 11 |
| Do you feel uncertain about what to do with your relative? | 0 | 2 | 0 | 0 | 1 | 1 | 0 | 0 | 0 | 0 | 4 |
| Do you feel you should be doing more for your relative? | 0 | 1 | 0 | 0 | 1 | 0 | 0 | 0 | 2 | 3 | 7 |
| Do you feel you could do a better job in caring for your relative? | 0 | 0 | 0 | 3 | 2 | 0 | 0 | 0 | 2 | 1 | 8 |
| Overall, how burdened do you feel in caring for your relative? | 1 | 4 | 0 | 1 | 2 | 1 | 2 | 0 | 2 | 0 | 13 |
|  |  |  |  |  |  |  |  |  |  |  |  |
| Total | 25 | 47 | 9 | 21 | 26 | 23 | 24 | 11 | 31 | 11 | 228 |
